# Dose to level I and II axillary lymph nodes and lung by tangential field radiation in patients undergoing postmastectomy radiation with tissue expander reconstruction

**DOI:** 10.1186/1748-717X-6-179

**Published:** 2011-12-28

**Authors:** James K Russo, Kent E Armeson, Ryan Rhome, Michele Spanos, Jennifer L Harper

**Affiliations:** 1 Department of Radiation Oncology, Hollings Cancer Center, Medical University of South Carolina: 169 Ashley Ave Room 168 MSC 318, Charleston, SC 29425, USA; 2 Division of Biostatistics and Epidemiology, Hollings Cancer Center, Medical University of South Carolina: 86 Jonathan Lucas St., Charleston, SC 29425, USA

**Keywords:** post-mastectomy radiation, axillary dose, tissue expander, breast reconstruction, tangent fields

## Abstract

**Background:**

To define the dosimetric coverage of level I/II axillary volumes and the lung volume irradiated in postmastectomy radiotherapy (PMRT) following tissue expander placement.

**Methods and Materials:**

Twenty-three patients were identified who had undergone postmastectomy radiotherapy with tangent only fields. All patients had pre-radiation tissue expander placement and expansion. Thirteen patients had bilateral expander reconstruction. The level I/II axillary volumes were contoured using the RTOG contouring atlas. The patient-specific variables of expander volume, superior-to-inferior location of expander, distance between expanders, expander angle and axillary volume were analyzed to determine their relationship to the axillary volume and lung volume dose.

**Results:**

The mean coverage of the level I/II axillary volume by the 95% isodose line (V_D95%_) was 23.9% (range 0.3 - 65.4%). The mean Ipsilateral Lung V_D50% _was 8.8% (2.2-20.9). Ipsilateral and contralateral expander volume correlated to Axillary V_D95% _in patients with bilateral reconstruction (p = 0.01 and 0.006, respectively) but not those with ipsilateral only reconstruction (p = 0.60). Ipsilateral Lung V_D50% _correlated with angle of the expander from midline (p = 0.05).

**Conclusions:**

In patients undergoing PMRT with tissue expanders, incidental doses delivered by tangents to the axilla, as defined by the RTOG contouring atlas, do not provide adequate coverage. The posterior-superior region of level I and II is the region most commonly underdosed. Axillary volume coverage increased with increasing expander volumes in patients with bilateral reconstruction. Lung dose increased with increasing expander angle from midline. This information should be considered both when placing expanders and when designing PMRT tangent only treatment plans by contouring and targeting the axilla volume when axillary treatment is indicated.

## Introduction

Mastectomy is a component of therapy for many women with breast cancer, both in the locally advanced and early stages. Recently, several institutions have reported increased mastectomy rates owing to several factors including MRI use, genetic testing, shifting patient preference and improved breast reconstruction options [1,2].

Post-mastectomy breast reconstruction rates have increased in the past 3 decades [3]. Options regarding timing of reconstruction include immediate, delayed, and most recently immediate-delayed. Currently, between 25-42% of women undergo immediate reconstruction and expander placement is a frequently utilized modality [4,5]. Delayed-immediate reconstruction using tissue expanders is an attractive option in patients requiring radiation because of the decreased complications and increased aesthetics associated with not radiating autologous tissue or a permanent implant [6,7].

Post-mastectomy radiation therapy (PMRT) is indicated in many of these patients to improve local control and overall survival [8-11]. As curability supersedes cosmesis in these patients, it is important to ask the question: "Does immediate reconstruction with tissue expanders impact the quality of PMRT?" Studies show that the unique geometry of immediate reconstruction using various reconstruction modalities can compromise coverage of the chest wall and internal mammary nodes and increase lung and heart dose [12-14]. These studies included few women with expanders and most patients had regional nodal irradiation.

Although axillary dose has been addressed in the breast-conservation setting it has not been studied in reconstructed patients. Expanders contain 20-30% more saline compared with the final implant. Therefore, it is important to analyze the population of women with tissue expanders. Recent attempts to eliminate axillary dissection with small numbers of positive nodes on sentinel node biopsy in early stage breast cancer are promising and do not appear to compromise outcomes in appropriately selected women who receive breast only radiation with tangents [15]. In light of this new paradigm, the dose delivered to the axilla is increasingly important as incidentally delivered axillary dose will be relied on to control potential residual disease. The purpose of this study is to define the dosimetric coverage of axillary volumes (level I/II) and the lung volume irradiated in patients treated with tangent fields following mastectomy and tissue expander placement and to identify variables that impact dose to these structures.

## Methods and Materials

Between 2006- 2010 a cohort of twenty-three patients who had undergone postmastectomy radiotherapy following tissue expander placement and expansion was identified. IRB approval was obtained for the study. Characteristics of the population are in Table 1. The study cohort had a tissue expander placed on the radiated side prior to radiation as part of a delayed-immediate reconstruction paradigm. Patients were excluded who had a reconstruction modality other than tissue expander such as an implant or autologous tissue without an expander. Patients with regional nodal radiation were allowed, however, these patients were replanned with tangents only. Therefore, no treatment plans incorporated a supraclavicular, internal mammary or posterior axillary boost field. None of the patients received a boost. Inverse-planned IMRT was not allowed.

**Table 1:** Patient and Treatment Characteristics

Characteristic		
Age (n = 23)		49 (25-71)*

Race		

	White	16

	AA	6

	Hispanic	1

Tumor Laterality		

	L	16

	R	4

	B/L	3

Tumor Location†		

	UOQ	9

	LOQ	2

	UIQ	4

	LIQ	2

	Chest Wall	1

	Overlapping	4

	Unknown	1

Histology		

	IDC	21

	ILC	2

Nodal Surgery Radiated Side		

	SLNB alone	4

	ALND	19

Type of Reconstruction		

	Ipsilateral Alone	10

	Bilateral††	13

Expander Location		

	Sub Pec Major	33

	Sub Latissimus Flap§	2

	Unknown	1

Clinical Stage		

	Tis||	3

	IA	5

	IIA	7

	IIB	7

	IIIA	2

	Unknown	2

Pathologic Stage		

	ypTis	1

	pTis	2

	ypT0N0	4

	ypIA	3

	ypIIA	2

pIIA		3

	ypIIB	2

	pIIB	5

	ypIIIA	3

	pIIIA	1

Systemic Therapy		

	Neoadjuvant	13

Adjuvant		10

* Median (range)

† Irradiated side only

†† 3 for contralateral synchronous primary and 10 prophylactically

§ 1 on irradiated side, 1 contralateral

|| All Tis had contralateral invasive disease

All patients underwent CT simulation from the mandible through the lungs using 3 mm slices in the supine position with an indexed breast board. No patient had their ipsilateral or contralateral expander deflated prior to or during radiation therapy. Tangents were designed to incorporate the chest wall with expander. In no patient were the tangents designed to treat the axilla. Field borders were initially defined on the CT simulation and were as follows: superior - base of clavicular head, inferior - 2 cm below the contralateral breast, medial - mid-sternum, lateral - mid-axillary line or appropriately lateral to insure adequate coverage of the most lateral extent of the expander. In case of bilateral reconstruction, the inferior border was placed in the area reasonably thought by the treating physician to include the pre-mastectomy breast extent.

Treatment planning was performed using step-and-shoot forward-planned IMRT using opposed tangential beams. A field-in-field technique was used to achieve dose homogeneity of 98 - 105% within the CTV. Half-beam blocks were used to prevent beam divergence into the lung. The prescription was normalized to a point just superficial to the surface of the pectoralis major in a plane perpendicular to the central axis at mid-separation. The prescription was delivered to the isodose line which best covered the breast CTV. This line was usually the 98-99% isodose line. The tangent angles were designed to include a minimum of lung tissue while maximizing coverage of the CTV.

**Figure 1:**
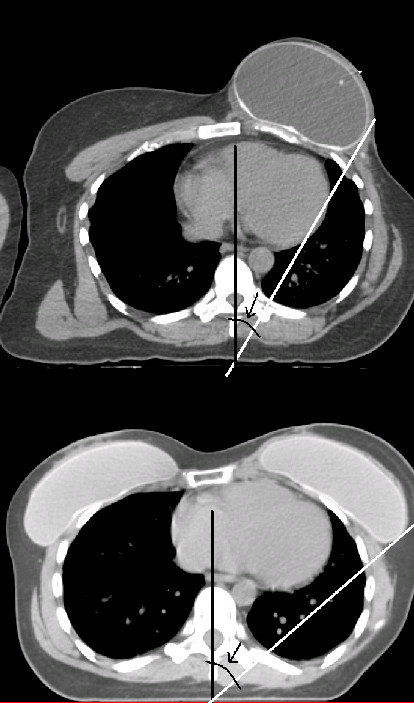
**Measurement of expander angle**. Lateral location of the expander defined by the expander angle (black arrow) measured on the axial slice with the most lateral expander location from midline to the lateral expander border in patient with a small (30° - top) and large (48° - bottom) angle.

Axillary levels I and II were contoured retrospectively using the RTOG contouring atlas [16]. Dose distributions to volumes of interest were determined using dose volume histograms. The following variables were examined to determine their relationship to the axillary V_D95% _and lung V_D50%_: Superior-to-inferior location of expander, expander angle, intra-expander distance expander volume and axillary volume. The superior-to-inferior location of the expander was defined as the distance between the inferior border of the humeral head and the superior border of the expander. To normalize this to patient height, the distance between the humeral head and the tip of the xiphoid process was also recorded. The expander angle was defined as the angle between the patient's sagittal midline and the most lateral border of the expander in the axial plane (Figure 1). Intra-expander distance was only recorded in patients with bilateral reconstruction and was defined as the closest distance between the most medial portions of the expanders. Lung V_D50% _was chosen because the 50% isodose line corresponds to the medial border of the half-beam blocked treatment fields, thus it should correlate with variables that change the treatment geometry. Also, the median prescription dose was 50 Gy; therefore the 50% isodose line corresponds to 25 Gy which is approximately the same as 20 Gy which has been shown to correlate with pneumonitis rates.

Summary statistics describe the various patient characteristics. Pearson correlation coefficients and their respective p-values were used to evaluate the relationship between the axillary V_D95%_, lung V_D50%_, and patient and expander physical characteristics.

## Results

Patient, tumor, and treatment characteristics are shown in table 1. The median patient age was 49. Nineteen patients had a full axillary dissection while 4 had sentinel node biopsy alone. Thirty-six breasts were reconstructed in 23 patients - 13 patients with bilateral procedures and 10 ipsilateral only. The most common type of reconstruction was a subpectoral expander occurring in 30 breasts. Two patients had the expander placed below a pedicled latissimus myocutaneous flap. All patients received chemotherapy, 57% received it neoadjuvantly and 43% adjuvantly.

**Figure 2:**
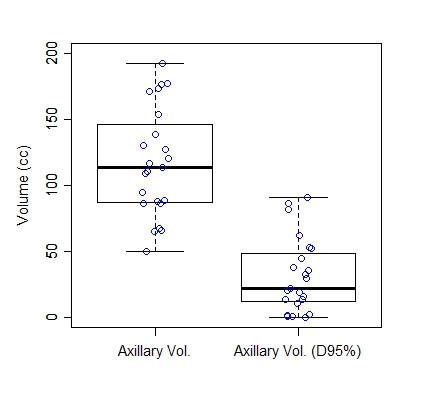
Box and whisker plots of the Axillary Volume and Axillary V_D95%_

**Figure 3:**
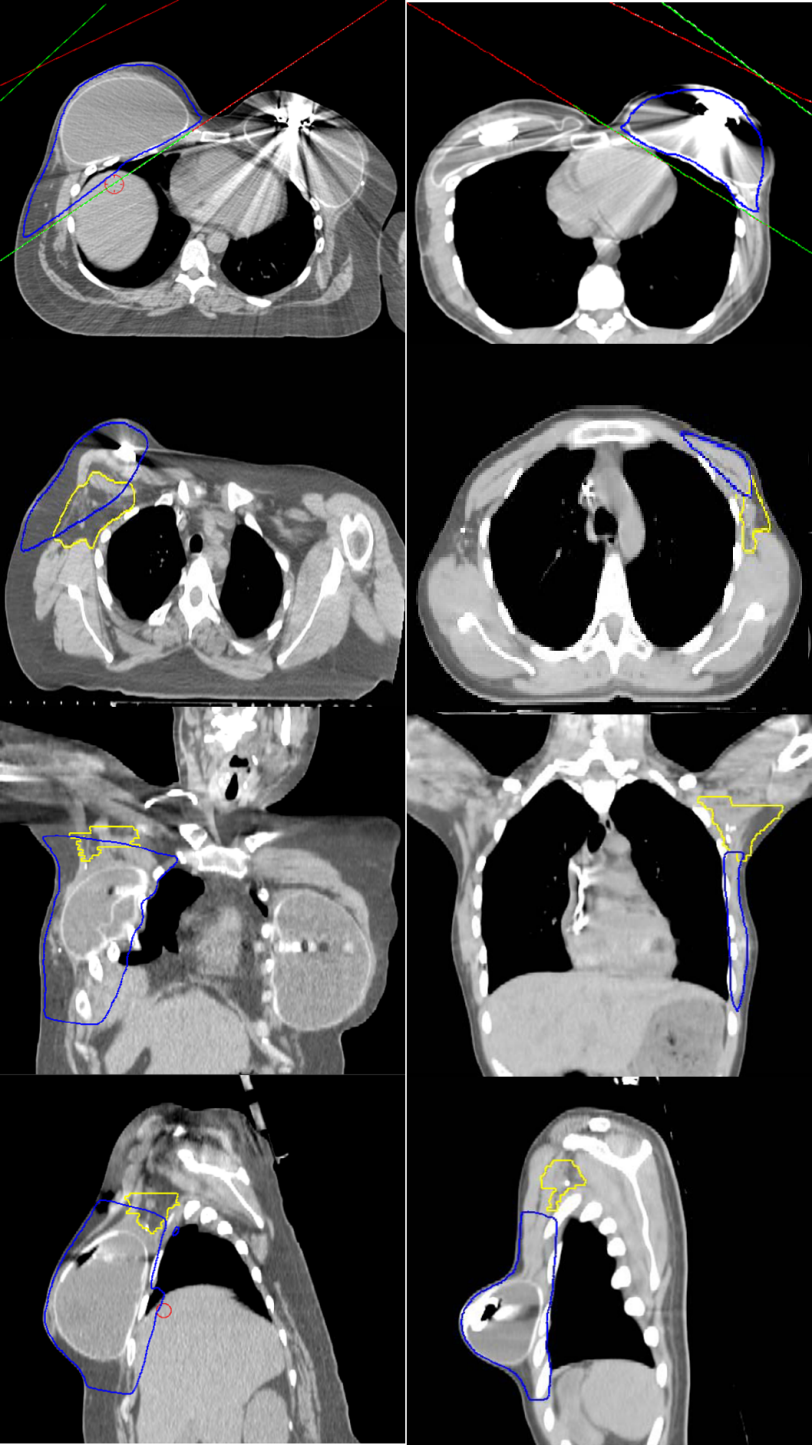
**Axillary coverage**. Coverage by the 95% isodose line as it relates to the axilla in a patient with 65% (left) and a patient with 3% (right) axillary coverage.

Table 2 shows descriptive statistics of the metrics that were analyzed. The mean level I/II axillary volume was 117.6 cm^3 ^(range 49.7 - 192.9 cm^3^) and the mean volume of the level I/II axilla covered by the 95% line was 31.6 cm^3 ^(range 0.4-90.6 cm^3^) (Figure 2). No patient had complete coverage of the level I/II axillary volume by the 95% isodose line. The mean percent of the level I/II axillary volume covered by the 95% isodose line was 23.9% (range 0.3-65.4%). Inspection of the isodose curves revealed that underdosing mainly occurred in the posterior-superior axilla as shown in Figure 3. The mean percent of the ipsilateral lung receiving 50% of the prescription dose was 8.8% (range 2.2-20.9%).

**Table 2:** Descriptive statistics

	Range	Median	Mean	StdDev
Axillary Volume*	49.7-192.9	113.6	117.6	41.4

Axillary V_D95%_†	0.4-90.6	21.9	31.6	28.1

% Axillary V_D95% _††	0.3-65.4	24.6	23.9	17

Expander Volume	170-492	437.4	485.4	190.4

Intra-Expander Distance	1.6-13.3	5.8	6.1	2.9

Expander Angle	30-49°	41°	41°	5.2°

Ipsilateral Lung V_D50%_	2.2-20.9	7.9	8.8	4.2

* All volumes in (cm^3^)

† Volume that receives at least 95% of the Rx dose

†† Percent of axilla that receives at least 95% of the Rx dose

The volume of both the ipsilateral and contralateral expander and the level I/II axillary volume correlated with % axillary V_D95% _(Table 3). The volume of the ipsilateral expander in all patients correlated to axillary V_D95% _with an r = 0.51 (p = 0.012). In the thirteen patients with bilateral reconstruction, the axillary V_D95% _correlated with the ipsilateral and contralateral expander volume (p = 0.01 and 0.006 respectively). However, in patients with ipsilateral reconstruction only, the axillary V_D95% _did not correlate with the expander volume (p = 0.60). The contralateral expander volume correlated to the ipsilateral expander volume with an r of 0.95. The superior-to-inferior position of the expander as measured from the humeral head did not correlate to the axillary dose. This also held true when this distance was normalized to the patient's thoracic size using the humeral head to xiphoid process distance. Neither the distance between expanders nor the expander angle impacted axillary V_D95%_.

**Table 3:** Pearson correlation (r) and p values of variables with axillary and lung dose

	r value: % Axillary V_D95%_	p value	r value: % Ipsilateral Lung V_D50%_	P value
HH to Expander	0.13	0.56	0.005	0.98

HH to Exp/HH to XP	0.16	0.46	0.03	0.88

Expander Angle	-0.25	0.24	0.41	0.05

Expander Distance	0.34	0.28	0.50	0.10

Ipsi Exp Volume	0.51	0.012	0.01	0.95

Ipsi Exp Vol*	0.18	0.60	0.21	0.54

Ipsi Exp Vol†	0.70	0.01	0.10	0.77

Contralat Exp Volume	0.76	0.006	0.19	0.56

Axillary Volume	0.52	0.011	N/A	N/A

* Only patients with ipsilateral expanders

† Only patients with bilateral expanders

Regarding the dose to the lung, only the expander angle correlated with the lung dose. Neither the superior-to-inferior expander position nor the volume of the expanders impacted lung dose. Axillary V_D95% _did not correlate with the Lung V_D50% _(r = 0.19).

## Discussion

In this study, we found that in patients undergoing post-mastectomy radiation therapy and delayed-immediate reconstruction with an inflated expander, the coverage of the axilla is impacted by the volume of the expander, but only in patients with bilateral reconstruction. The underdosing occurred primarily in the posterior-superior extent of axillary levels I and II. The inferior portion of the axilla is difficult to define radiographically in this population of patients. Also, dose to the lung is correlated with the angle of the expander. The superior-to-inferior extent of the expander did not impact the axillary or lung dose.

A possible explanation for the correlation of axillary coverage and expander volume in bilaterally reconstructed patients is that the expander was used as a surrogate for the pre-mastectomy breast tissue because there is no contralateral native breast to use as a template. Thus, if larger volume expanders are placed, then the tangents would be designed to completely encompass these larger expanders and the amount of axillary volume incidentally included in the tangents would increase. Conversely, if smaller volume expanders were placed, then the tangents would conform to the size of the expander and the amount of axillary volume incidentally included in the tangents would decrease.

A relationship was also seen between the axillary volume and the axillary V_D95%_. This may be due to the difficulty in delineating the inferior extent of axillary level I in this population of patients. The RTOG contouring atlas defines the inferior extent of level I as the insertion of the pectoralis major into the ribs [16]. We found this insertion point difficult to locate due to disruptions in the normal anatomy from the expanders' subpectoral location and also that an alloderm graft was often used in the inferior-lateral portion of the expander to hold it in place on the chest wall.

The only parameter in our study that impacted lung dose was the lateral location of the expander as measured by the angle of the expander from midline sagittal plane. This is likely because deeper tangents are required to treat the more lateral expanders thus resulting in a higher lung dose. As modern systemic agents used in breast cancer can be associated with a small increased risk of pneumonitis, this should be taken into account when placing expanders. Also, the risk of secondary pulmonary malignancies, especially in smokers, may be reduced by minimizing exposure of the lung [17,18].

Our study findings of poor axillary coverage relative to the prescription dose agree with data from several investigators. Aristei et al showed that the median D_90 _of levels I and II was 6.75 Gy and 1.75 Gy, respectively, in breast conserved patients treated with tangents only undergoing 2D simulation with 3D dose analysis [19]. This represented, as a percent of the prescription dose of 50 Gy, a median D_90 _of 13.5% to level I and 3.5% to level II. Smitt et al showed that in patients with conserved breasts undergoing CT planning with the goal of covering the breast alone, a mean axillary dose of more than 90% of the prescription dose was only achieved in 1 of 6 patients [20]. Also, underdosing occurred in the posterior-superior portion of levels I and II of the axilla as in our study. Our data are consistent with McCormick et al who reported that axillary hemostasis clips in 45 patients are included in 2D planned tangents on 38% of patients and that incomplete clip coverage occurred in the posterior-superior portion [21]. Krasin et al reconstructed 2D plans to obtain 3D dose-volume data in patients treated with standard breast tangents and found that out of 25 patients, one had more than 95% coverage of level I and none had more than 95% coverage of level II. Also, the mean V_20 _was 7.5% which is slightly less than our 8.8% mean amount of lung receiving 50% of the prescription dose of 50 Gy [22].

Published data also conflicts with our results. Goodman et al reported that in patients undergoing tangential only radiation, levels I and II were covered in 8 of 9 patients [23]. Details regarding anatomic boundaries of the axillary were not provided. However, the anatomic landmarks for contouring the axilla likely differed from our study as the RTOG contouring atlas had not been published. Also, the surgical therapeutic and reconstructive management of these patients varied.

In a similar investigation to ours, Reed et al performed an analysis of axillary levels I and II dose using 3D planning in patients with breast conservation and found that a mean of 55% of the axilla was covered by the 95% line which is more than double our finding of 23.9% [24]. Reasons for this difference are that axillary contouring landmarks were different which could lead to more generous contouring in well covered areas like low level I and less generous in poorly covered areas such as in the posterior-superior direction, an area often underdosed in our study. Indeed the mean axillary volume in their study was 146.3 cm^3 ^compared with 117.6 cm^3 ^in ours. However, similar to our study the posterior-superior area was not well covered. Second, these patients were not planned with step-and-shoot techniques as in our study and it is possible that more traditional breast only irradiation techniques deliver a higher dose to the axilla.

A limitation of our study is the small number of patients. Nonetheless, to our knowledge it represents the largest study of axillary and lung doses in delayed-immediate reconstructed women undergoing modern radiotherapy, many of whom had bilateral reconstruction.

Our results are important for several reasons. Recent studies have shown that both mastectomy rates and reconstruction rates are increasing [1-3]. As such we must analyze this group of post-mastectomy women with respect to delivery of radiation and outcomes. In addition, the results of our study have implications in light of the recently reported ACOSOG Z0011 trial which showed that women with less than 3 positive nodes on sentinel node biopsy who received tangent only radiation had overall survival, locoregional control and disease-free survival that is not inferior to those who received axillary node dissection and the same radiation [15]. Although in this trial women received breast conservation and not mastectomy, as in our study, the results may be applicable to women who choose mastectomy but were otherwise eligible for the trial. As these women may decide to pursue post-mastectomy reconstruction, it is important to quantify the dose to the axilla. This is particularly important considering 27.3% of women on the ACOSOG trial with positive sentinel nodes had additional metastatic nodes on subsequent axillary dissection.

## Conclusions

Efforts to reduce morbidity by decreasing the extent of axillary dissection must be accompanied by comprehensively quantifying dose and factors that impact dose to the axilla in all subgroups of patients, including those with tissue expanders. This information will be helpful both for recurrence and morbidity endpoints. Our study is the first report of axillary dose in women with tissue expanders undergoing breast only irradiation. This analysis suggests that in patients undergoing PMRT with tissue expanders in place, incidental doses delivered by opposed tangents to the Level I and II regions, as defined by the RTOG contouring atlas, do not provide adequate dosimetric coverage of these regions. This should be considered when designing PMRT treatment fields. However, the clinical significance of underdosing this volume is unknown. In addition, the increased lung dose associated with the lateral expander location should be taken into consideration when placing expanders. How axillary and lung coverage affects endpoints such as axillary recurrence, pulmonary toxicity, locoregional failure, distant metastasis and morbidity remains to be seen and should be the subject of future investigations.

## Competing interests

The authors declare that they have no competing interests.

## Authors' contributions

JR carried out the data collection, assisted in the statistical analysis and drafted the manuscript, KA performed the statistical analysis, RR assisted in data collection and manuscript drafting, MS assisted in data collection, JH conceived of the study and assisted in drafting of the manuscript. All authors have read and approved the final manuscript.

## Author information

Dr. Harper is a radiation oncologist, certified by the American Board of Radiology. Her primary focus is in the treatment of breast cancer, palliative care, and head and neck cancer. In the field of breast cancer, Dr. Harper has published extensive clinical research on the topic of accelerated partial breast irradiation. She is a member of Hollings Cancer Center Protocol Review Committee,

Hollings Cancer Center Cancer Registry Data Reviewer and Southwest Oncology Group Breast Cancer Committee Member.

Dr Russo is a resident in radiation oncology in his 4^th ^year of training. He has an interest in breast cancer research and has published in this area as well as CNS, lung and GYN.
